# Variation in characterization and probiotic activities of polysaccharides from litchi pulp fermented for different times

**DOI:** 10.3389/fnut.2022.993828

**Published:** 2022-08-24

**Authors:** Chunmei He, Ruifen Zhang, Xuchao Jia, Lihong Dong, Qin Ma, Dong Zhao, Zhida Sun, Mingwei Zhang, Fei Huang

**Affiliations:** ^1^College of Food Science and Technology, Huazhong Agricultural University, Wuhan, China; ^2^Sericultural and Agri-Food Research Institute Guangdong Academy of Agricultural Sciences/Key Laboratory of Functional Foods, Ministry of Agriculture and Rural Affairs/Guangdong Key Laboratory of Agricultural Products Processing, Guangzhou, China

**Keywords:** litchi, fermentation, polysaccharide, chemical structure, probiotic activity

## Abstract

This study investigated the chemical structures and probiotic potential of different polysaccharides (LPs) extracted from the litchi pulp that fermented with *Lactobacillus fermentum* for different times (i.e., 0–72 h corresponding to LP-0 through LP-72, respectively). Fermentation times affected the yields, total sugar contents, uronic acid contents, molecular weights, and monosaccharide compositions of LPs. The LPs yields and uronic acid contents exhibited irregular trends in association with fermentation time, while total sugar contents decreased, and the molecular weights increased. Particularly, LP-6 contained the highest extraction yields (2.67%), lowest uronic acid contents, and smallest average Mw (104 kDa) (*p* < 0.05). Moreover, analysis of the monosaccharide composition in the fermented LPs indicated that the proportions of glucose decreased, while arabinose and galacturonic acid proportions increased relative to unfermented LP-0. Further, LP-6 demonstrated the highest growth for *Bifidobacterium* compared to LP-0, while the other fermentation time led to comparable or worse probiotic promoting activities. These results suggest that lactic acid bacteria fermentation alters the physicochemical properties of litchi polysaccharides, such that suitable fermentation time can enhance their probiotic activities.

## Introduction

Litchi (*Litchi chinensis Sonn*.) is a tropical to subtropical fruit that has become one of the most popular fruits because of its high nutritive value (1). Litchi is prone to spoilage and has a short shelf-life due to its rich nutrition content and maturation during high temperature and high humidity conditions. Processing is an important way to prolong litchi shelf-life. Lactic acid bacteria fermentation is widely used in fruit processing, because it enhances fruit nutritional properties and improves flavors (2, 3), thereby representing a useful means of exploiting litchi nutritional content. For example, the total phenolic and total flavone contents in litchi juice dramatically increase after *Lactobacillus. casei* fermentation (4). The contents of total amino acids in litchi juice-soybean protein complexes decrease with lactic acid bacteria fermentation (5), while the species and contents of fatty acid groups increase (6). Additionally, *Lactobacillus plantarum* HU-C2W was used as a starter culture and enhanced the production of γ-aminobutyric acid in fermented litchi juice (7). Overall, investigations of lactic acid bacterial fermentation of litchi have primarily focused on changes in nutrient content, like those of polyphenols, flavonoids, fatty acids, and γ-aminobutyric acid. In contrast, few studies have evaluated the effects of lactic acid bacteria fermentation on the primary functional ingredients in litchi pulp. Previous studies have suggested that numerous health benefits of litchi pulp could be related to polysaccharides that exhibit immunostimulatory (8), antioxidant (9, 10), and antiproliferative effects (11). However, the effects of lactic acid bacterial fermentation on litchi pulp polysaccharides were unknown yet.

Previous studies showed that polysaccharides are indigestible food components and exhibit their bioactivities primarily depending on the regulation of intestinal flora and their metabolites (12). For example, *Lycium barbarum* polysaccharides significantly increase the abundances of some potential probiotic bacterial genera like *Akkermansia*, *Lactobacillus*, and *Prevotellaceae* that can promote immunostimulatory activity (13). In addition, the anti-diabetic effects of *Plantago asiatica* L. polysaccharides in type 2 diabetic rats may be associated with increased colon bacterial diversity and abundances, including those of *Lactobacillus fermentum* and *Prevotella loescheii* (14). Further, bitter gourd polysaccharides have been shown to improve intestinal flora disorders and increase the abundance of beneficial flora that can ameliorate rat obesity (3). Thus, polysaccharides exert health benefits by selectively promoting intestinal probiotic strains. Hence, an assessment of the probiotic activities of polysaccharides is essential for evaluating their potential health effects. Previous studies have reported that polysaccharides from pistachio hulls exhibit probiotic potential by promoting the proliferation of *L. plantarum* PTCC 1896 and *L. rhamnosus* GG (15). Similarly, yam polysaccharides have been shown to significantly promote *S. thermophilus* growth (16). Longan polysaccharides significantly improve *Leuconostoc mesenteroides* and *Lactobacillus casei* proliferation (17). In contrast, the potential probiotic activities of litchi polysaccharides remain unknown.

The present study aimed to investigate the influence of lactic acid bacterial fermentation on the physicochemical properties and probiotic activities of litchi pulp. To accurately identify changes in polysaccharide structures and prebiotic properties during fermentation, polysaccharides were prepared from different fermentation times (0, 6, 12, 24, 36, 48, 60, and 72 h), followed by the analysis of their chemical composition, monosaccharide composition, molecular weights (Mw), functional group characteristics, and their promotion of *Bifidobacteria* strain proliferation.

## Materials and methods

### Materials and chemicals

#### Plant materials and chemicals

Fresh litchi (cv. Hei-ye) fruits were provided by the Pomology Research Institute of Guangdong Academy of Agricultural Sciences (Guangzhou, China). Fresh litchi pulp was dried under hot air at 70°C for further analysis.

Standard dextran, rhamnose (Rha), arabinose (Ara), glucose (Glu), galactose (Gal), and mannose (Man) chemicals were purchased from Sigma (St. Louis, MO, United States). All other reagents were of analytical grade. Man-Rogosa-Sharpe (MRS) and sugar-free MRS were purchased from Guangdong Huankai Microbial Technology Co., Ltd. (Guangzhou, China). All other chemicals were purchased from Guangzhou Qiyun Biological Co., Ltd. (Guangzhou, China) and were of analytical grade.

#### Bacterial strains

*Lactobacillus fermentum* CICC 21828 was purchased from the China Center of Industrial Culture Collection (Beijing, China). *Bifidobacterium longum* ATCC 15707, *Bifidobacterium infantis* GDMCC 1.207 and *Bifidobacterium adolescentis* GDMCC 1.278 were purchased from the Guangdong Microbial Culture Collection Center (Guangzhou, China). Strains were stored in MRS broth containing 25% glycerol within liquid nitrogen until later use. Prior to use, bacterial strains were revived in MRS broth supplemented with 0.05% L-cysteine using previously described procedures (18).

### Preparation of fermented litchi pulp

Dried litchi pulp pH was adjusted to 5.0 ± 0.2 with 2 M NaOH after homogenizing with water (1:7 w/v) for 5 min. Litchi juice was then sterilized at 121°C and 103.4 kPa for 20 min. Individual Erlenmeyer flask was used for 100 mL of sterilized litchi juice without any other nutrients. Each flask received 1 mL inoculum containing 6.0 log CFU/mL of activated *L. fermentum*. Fermentation was then initiated by incubation on a reciprocal shaker for 72 h at 37°C. Three duplicate samples were taken at 0, 6, 12, 24, 36, 48, 60, and 72 h to determine culture pH and extract polysaccharides. Enumeration of *L. fermentum* cell densities at each fermentation time point were also conducted using MRS agar.

### Litchi polysaccharide extraction

Litchi polysaccharides were extracted as previously described (19), with slight modifications. Briefly, fermented litchi pulp was boiled for 10 min at 100°C to kill live *Lactobacillus*. Boiled juice was topped with 900 mL distilled water up to 1 L, followed by incubation at 90°C for 4 h before collecting filter liquor. The extraction process was repeated and the filtrates were collected, combined, and then vacuum concentrated at 65°C. To remove proteins, the extracted solutions were subjected to the Sevag method four times (20). Fourfold volumes of ethanol were added to the protein-removed solutions to achieve polysaccharide precipitation at 4°C for 24 h. The precipitates were collected by centrifugation at 4,000 × *g* for 10 min, washed with ethanol, and lyophilized to obtain the final litchi polysaccharides (LPs). The polysaccharides extracted from different time points during litchi pulp fermentation are referred as follows: LP-0, LP-6, LP-12, LP-24, LP-36, LP-48, LP-60, and LP-72, respectively.

### Physicochemical properties of litchi polysaccharides

#### Chemical compositions

Total sugar contents were determined using the phenol-sulfuric acid method according to previously published methods (21) and with glucose as the standard. Briefly, 1 mL crude LP solution (0.25 mg/mL) was mixed with 500 μL 6% phenol and 2.5 mL concentrated H_2_SO_4_. After cooling in an ice bath for 30 min, the absorbance of the sample was measured using a UV–VIS spectrophotometer at 490 nm. Uronic acid contents were determined using a modified hydroxy diphenyl method and with galacturonic acid as the standards (11). Briefly, 0.25 mL of the crude LP solution (0.25 mg/mL) was added with 1.5 mL sulfuric/tetraborate and vortexed. The mixture was in a water bath at 100°C for 5 min. After cooling in an ice bath, 25 μL m-hydroxydiphenyl reagent was added. Then the absorbance of the sample was measured using a UV–VIS spectrophotometer at 524 nm. Lastly, protein concentrations were measured with the Bradford method using bovine serum albumin for standards (22). Briefly, 1 mL of the crude LP solution (10 mg/mL) was added with 5 mL of Bradford reagent and incubated at 37°C for 15 min in a water bath. Absorbance was read at 590 nm in a UV-VIS spectrophotometer.

#### Molecular weight analysis

To determine the molecular weights of the polysaccharides, the average molecular weights (Mw) were detected by high-performance gel permeation chromatography (HPGPC) using an Agilent technologies 1260 series instrument (Agilent Co., United States) equipped with a Shodex OH-pak SB-804 HQ column (8 mm × 300 mm). Chromatographic procedures and conditions were performed as previously described (23). Dextran standards with different molecular weights (6.7 × 10^5^, 4.1 × 10^5^, 2.7 × 10^5^, 5 × 10^4^, 2.5 × 10^4^, 1.2 × 10^4^, 5 × 10^3^, and 1 × 10^3^ Da) were used to calibrate the standard curve using the GPC software program (Agilent Technologies, Inc., United States, version 3.4).

#### Monosaccharide compositions

Litchi polysaccharides monosaccharide compositions were determined by HPLC with PMP precolumn derivatization (24). Briefly, polysaccharide samples (2.0 mg) were hydrolyzed with 0.2 mol/L trifluoroacetic acid at 120°C for 2 h. After hydrolysis, excess acid was removed by evaporation under a nitrogen atmosphere. Sodium hydroxide (0.1 mol/L) was added to dissolve the dried hydrolyzates. The mixtures were treated with 0.5 mol/L PMP in methanol and then incubated at 70°C for 30 min. After cooling to room temperature, the mixture was neutralized by adding 0.3 mol/L hydrochloric acid and then extracted with chloroform, followed by chloroform. The extractions were repeated three times. The aqueous phases were filtered through a 0.22 μm membrane and the resulting solutions were analyzed on an Agilent 1100 HPLC system (Agilent, United States) with a C18 column (4.6 mm × 250 mm, 5 μm) and a DAD detector. Elution was performed with a mixture of 0.1 M phosphate buffer solution (pH 7.0) and acetonitrile in a ratio of 82:18 (v/v), a flow rate of 1.0 mL/min, and a detection wavelength set as 250 nm.

#### Fourier transform infrared spectroscopy

A Fourier Transform Infrared (FT-IR) spectrophotometer (NEXUS 670, Nicolet, United States) was used to investigate the functional groups within LPs. Ground LPs were mixed with dry KBr and pressed into a mold to generate a tablet that was then subjected to the spectral region of 4000–400 cm^–1^ (25).

### Probiotic activities

Three *Bifidobacterium* strains—*B. adolescentis*, *B. infantis*, and *B. longum* were used to investigate *in vitro* probiotic activities of the LPs isolated from the unfermented and fermented litchi pulps. Carbohydrate-free MRS broth supplemented with 0.05% (w/v) L-cysteine was used as the basal medium for the experiments and as the blank control, while fructooligosaccharide (FOS) was used for the positive control. All LPs and FOS were filter-sterilized and separately added to the basal medium to obtain 1.0% (w/v) final concentrations. Activated *Bifidobacterium* strains were added to medium at final concentration of 1 × 10^6^ CFU/mL and then incubated at 37°C for 48 h under anaerobic conditions (85% N_2_, 10% CO_2_ and 5% H_2_). Bacterial growth was monitored by measuring culture optical density at 600 nm (OD_600_) at 24 and 48 h, while culture pH was simultaneously measured using a pH meter (pH S-3C, Shanghai Precise Scientific Instrument Corp., China).

### Statistical analyses

All experiments and analyses were performed in triplicate. Data analysis was performed using the SPSS statistical software program (version 19; SPSS, Inc., Chicago, IL, United States). Results are reported as means ± SD. Data were subjected to one-way ANOVA tests followed by Duncan’s multiple range tests to identify statistical differences between values. Statistical significance was considered at *p* < 0.05.

## Results and discussion

### Litchi pulp fermentation

**FIGURE 1:**
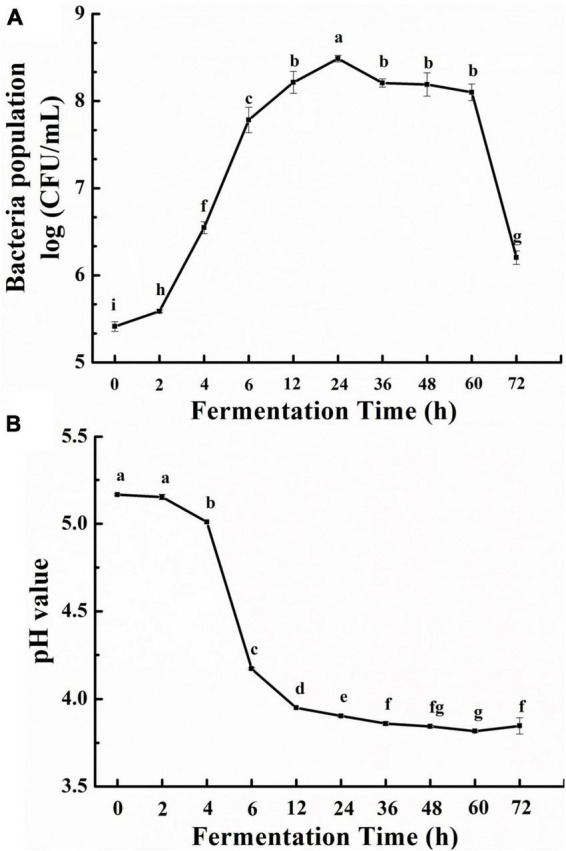
Growth effects of litchi pulp on *Lactobacillus fermentum* with different fermentation times **(A)**, and the pH of litchi pulp cultures **(B)**. Mean ± SD (*n* = 3). Different lowercase letters represent statistically significant differences among samples (*p* < 0.05).

The effects of fermentation time on the growth of *L. fermentum* and litchi pulp pH were shown in Figure 1, where it can be seen that bacterial counts increased during fermentation (Figure 1A). Specifically, an initial population of 5.41 log fu/mL, slightly increased during the lag phase (0–2 h), and then rapidly increased to 8.49 log CFU/mL during the logarithmic phase (2–24 h). During the stationary phase, bacterial number slowly decreased until 60 h (8.28 log CFU/mL), then markedly decreased from 60 to 72 h (6.20 log CFU/mL) in the decay phase due to nutrient deficiencies.

The increased bacterial number indicated that *L. fermentum* used the nutrients within the litchi juice to grow. The consumption of sugars by lactic acid bacteria results in the accumulation of lactic acid and short-chain fatty acids (23). Consequently, the growth of probiotic microorganisms was also accompanied by decreased medium pH. Accordingly, the pH of litchi juice decreased throughout fermentation. Specifically, pH began at 5.16, slightly dropped over 2–4 h, then quickly fell to 3.95 at 12 h. And pH slowly continued to decline until reaching 3.81 at 60 h (Figure 1B).

### Preliminary characterization of litchi polysaccharides

The yields and contents of total sugar, uronic acids, proteins, in addition to the molecular weights of isolated LPs were evaluated (Table 1). Compared with unfermented samples, polysaccharide yields first increased with fermentation at 6 h, then decreased with increasing fermentation time from 6 to 48 h, and then gradually increased in later fermentation times (48–72 h). LP-6 exhibited the highest yield among all samples (*p* < 0.05). The total sugar content of non-fermented litchi pulp polysaccharide was higher than those of fermented samples (*p* < 0.05). Similar decreases in carbohydrate contents have also been observed for polysaccharides from rice bran after *Grifola frondosa* fermentation (26). In addition, LP-72 exhibited the highest uronic acid content, while LP-6 exhibited the lowest (*p* < 0.05). The protein contents in fermented polysaccharides decreased with fermentation time (*p* < 0.05). Further, the protein contents in non-fermented polysaccharides were lower than in fermented samples (i.e., LP-6, LP-12, LP-24, LP-36, and LP-48) (*p* < 0.05).

**TABLE 1:** Physicochemical properties of polysaccharides derived from litchi pulp fermented with *Lactobacillus fermentum* for different times.

LPs	Extraction yield (%)	Total sugars (%)	Uronic acid (%)	Protein (%)	Mw (× 10^4^ Da)
LP-0	1.73	65.27 ± 0.01^a^	33.33 ± 0.93^bc^	0.24 ± 0.01^e^	13.60
LP-6	2.67	61.90 ± 0.00^b^	20.87 ± 0.88^e^	1.03 ± 0.02^b^	10.40
LP-12	1.90	56.92 ± 0.01^cd^	31.87 ± 0.86^bc^	1.28 ± 0.01^a^	13.80
LP-24	1.93	52.49 ± 0.01^e^	24.84 ± 0.30^d^	1.29 ± 0.01^a^	14.50
LP-36	1.50	59.18 ± 0.01^bc^	33.52 ± 0.04^b^	0.61 ± 0.01^c^	14.80
LP-48	1.66	53.21 ± 0.01^e^	30.51 ± 0.05^c^	0.40 ± 0.01^d^	16.00
LP-60	2.08	54.66 ± 0.04^de^	24.03 ± 0.10^d^	0.01 ± 0.01^f^	15.90
LP-72	2.34	55.45 ± 0.01^de^	36.67 ± 0.66^a^	0.02 ± 0.01^f^	15.20

Different lowercase letters represent statistically significant differences among samples (*p* < 0.05).

The Mw of LPs were determined using GPC with RID. Overall, LPs Mw first decreased and then increased with fermentation time, while LP-6 exhibited the lowest Mw (Table 1). Similar results have also been observed for polysaccharide from longan pulp after fermentation for different times (19). The prominent decrease in Mw of LP-6 could be related to the accumulation of carbohydrases secreted by bacteria during the lag and logarithmic phases (Figure 1A). Such activities would lead to the efficient hydrolysis of polysaccharides, thereby reducing overall Mw values and providing useable carbon sources for bacterial growth (27). However, the Mw of the fermented polysaccharides (except for LP-6) increased to various degrees compared to the LP-0 samples. The increased Mw likely arose from that the bacteria preferentially use the smaller Mw fractions over larger Mw fractions (28), thereby increasing the average Mw across fermentations over time.

Monosaccharide compositional analyses revealed that all LPs were heteropolysaccharides that comprised different ratios of galacturonic acid, glucose, arabinose, galactose, mannose, glucuronic acid, xylose, and/or rhamnose (Table 2). The monosaccharide compositions of the seven fermented polysaccharides were similar to those of unfermented polysaccharide, indicating that fermentation did not change the essential monosaccharide types identified within the litchi polysaccharides. However, the ratios of monosaccharide compositions considerably differed. The major monosaccharides in the LPs were galacturonic acid, glucose, and arabinose. Galacturonic acid was most abundant in all samples, with a minimum value of 38.79% in LP-24 and a maximum value of 48.55% in LP-12. The relative percentages of glucose in the other fermented LPs (except for LP-6) were lower than in the non-fermented LP-0 sample. Conversely, the percentage compositions of arabinose and galactose exhibited opposite trends. This was consistent with the other reported finding, as *Saccharomyces cerevisiae* and *Bacillus subtilis* fermentation decreased the molar ratio of glucose among wheat bran polysaccharides, while increasing the molar ratio of arabinose and galactose (24).

**TABLE 2:** Monosaccharide compositions of polysaccharides derived from litchi pulp fermented with *Lactobacillus fermentum* for different times.

LPs	GalA (%)	Glu (%)	Ara (%)	Gal (%)	Man (%)	GlcA (%)	Xyl (%)	Rha (%)
LP-0	40.45 ± 5.01^ab^	30.14 ± 3.83^a^	18.72 ± 1.46^ab^	5.35 ± 0.01^ab^	2.59 ± 0.01^c^	2.42 ± 0.21^b^	0.17 ± 0.24^a^	0.17 ± 0.24^a^
LP-6	39.24 ± 0.25^a^	33.41 ± 1.85^a^	17.79 ± 1.77^a^	5.04 ± 0.17^a^	2.22 ± 0.08^a^	1.95 ± 0.19^a^	0.34 ± 0.06^abc^	–
LP-12	48.04 ± 1.76^c^	20.37 ± 0.50^bc^	20.80 ± 0.67^bc^	5.85 ± 0.03^c^	2.18 ± 0.04^a^	2.35 ± 0.04^b^	0.25 ± 0.36^ab^	0.15 ± 0.22^a^
LP-24	38.79 ± 2.00^a^	30.57 ± 2.05^a^	19.74 ± 0.03^abc^	5.49 ± 0.19^b^	2.27 ± 0.08^a^	2.37 ± 0.10^b^	0.51 ± 0.09^abc^	0.26 ± 0.00^a^
LP-36	46.63 ± 4.48^bc^	22.02 ± 2.85^bc^	19.71 ± 1.47^abc^	6.16 ± 0.13^c^	2.42 ± 0.02^b^	2.50 ± 0.17^bc^	0.39 ± 0.05^abc^	0.15 ± 0.21^a^
LP-48	44.79 ± 0.12^abc^	18.66 ± 0.56^c^	23.53 ± 0.58^de^	6.69 ± 0.12^d^	2.50 ± 0.06^bc^	2.86 ± 0.04^cd^	0.67 ± 0.01^bc^	0.30 ± 0.00^a^
LP-60	38.82 ± 2.78^a^	28.18 ± 1.53^ab^	21.56 ± 0.64^cd^	6.03 ± 0.12^c^	2.17 ± 0.02^a^	2.57 ± 0.20^bc^	0.54 ± 0.09^abc^	0.13 ± 0.18^a^
LP-72	43.91 ± 1.90^abc^	17.82 ± 0.50^c^	24.29 ± 0.94^e^	7.45 ± 0.27^e^	2.48 ± 0.03^bc^	3.14 ± 0.18^d^	0.74 ± 0.21^c^	0.17 ± 0.24^a^

Different lowercase letters represent statistically significant differences among samples (*p* < 0.05).

**FIGURE 2:**
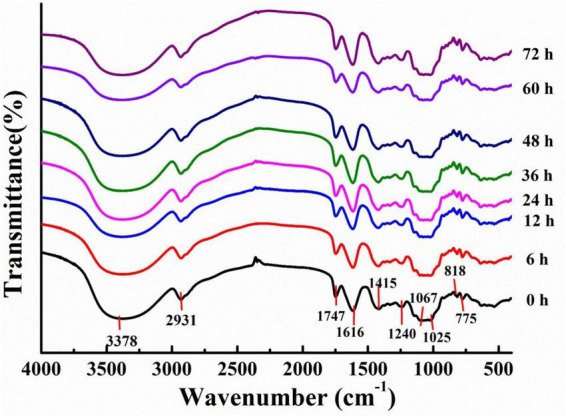
Fourier Transform Infrared (FT-IR) spectra for polysaccharides derived from litchi pulp fermented with *Lactobacillus fermentum* for different times.

Fourier Transform Infrared analysis of LPs was also conducted (Figure 2). All LPs fractions exhibited a strong broad peak at 3600–3200 cm^–1^ and a sharp weak band at 2930–2926 cm^–1^ in their FT-IR spectra, which arose from the O–H and C–H stretching vibrations, respectively (29). In addition, characteristic absorption peaks associated with uronic acid were observed at 1747 and 1616 cm^–1^ (30), while a characteristic absorption peak for galacturonic acid in pectic polysaccharides was observed at 1025 cm^–1^ (31). The observed absorption peaks near 1100–1000 cm^–1^ derived from characteristic C–O–C glycosidic bond vibrations and ring vibrations overlapping glycosidic bridges, indicating the presence of pyranose (32). Absorption signals near 890 cm^–1^ and 775 cm^–1^ were attributed to β– and α– type glycosidic linkages, respectively (33, 34). Together, these results provided important insights into potential LPs that represented acidic heteropolysaccharides with both α– and β– ring structures. The characteristic absorption peaks of LPs were similar, demonstrating that fermentation did not alter the primary functional group compositions of polysaccharides (35).

### Litchi polysaccharides probiotic activity

**FIGURE 3:**
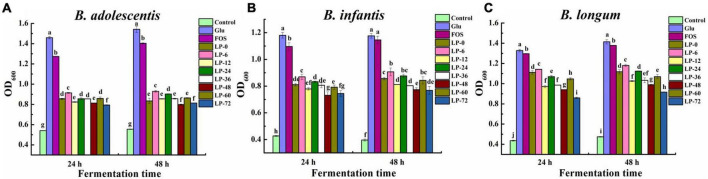
The growth of probiotic strains *Bifidobacterium adolescentis* **(A)**, *Bifidobacterium infanitis* **(B)**, and *Bifidobacterium longum* **(C)** cultivated on polysaccharides derived from litchi pulp fermented with *Lactobacillus fermentum* for different times. Mean ± SD (*n* = 3). Different letters indicate significant (*p* < 0.05) differences at the same incubation time.

Bacterial growth of three *Bifidobacterium* strains was evaluated when LPs, Glu, or FOS were used as carbon sources for 24 and 48 h incubation (Figure 3). In the case of *B. adolescentis*, all evaluated carbon sources supported significant growth of probiotic strain cultures when compared against the blank control (Figure 3A). This suggested LPs could be used by probiotic strains for growth similarly to FOS and Glu, and this was supported by previous findings (36). Bacterial growth dynamics were similar between 24 and 48 h and increased following the order of Glu >FOS >LPs at both fermentation time points (*p* < 0.05). Moreover, some fermented LPs (i.e., LP-6, LP-24, and LP-60) exhibited better probiotic activity at 48 h compared to unfermented polysaccharides (LP-0), with LP-6 exhibiting the highest microbial growth promotion.

*Bifidobacterium infantis* growth for both Glu and blank groups did not exhibit any obvious changes between different incubation times, while LPs and FOS cultures exhibited marginal increases at 48 h compared to 24 h (Figure 3B). This result was consistent with a previous study (30) that the complex structures of LPs and FOS likely led to their slower metabolism rate and continuous probiotic growth at 48 h relative to Glu. Moreover, differences in bacterial growth on fermented litchi polysaccharides were exhibited compared to the unfermented control. LP-6 and LP-24 exhibited better probiotic activity compared to LP-0, while LP-6 activity was the highest at 48 h. The highest OD_600_ values of the LPs groups were generally observed at 48 h, with relative differences in the following order of LP-6 > LP-0 ≈ LP-24 ≈ LP-60 > LP-12 ≈ LP-36 > LP-48 ≈ LP-72.

The effects of LPs on the growth of *B. longum* strains were also evaluated (Figure 3C). All LPs, FOS and Glu supplements stimulated greater bacterial activity compared to the blank control. Bacterial growth increased in all test groups at 48 h relative to 24 h. The LP-6 exhibited the most bacterial growth among all LPs, while the LP-72 exhibited the worst growth (*p* < 0.05) at both 24 and 48 h. Further, only LP-6 exhibited the most bacterial growth among fermented LPs compared to unfermented LP-0, while the other fermented LPs exhibited comparable or worse growth of bacteria. The maximum OD_600_ values for LPs cultures at 48 h followed the order of LP-6 > LP-0 ≈LP-24 > LP-60 > LP-36 ≈ LP-12 > LP-48 > LP-72. Overall, these results indicated that the fermentation of litchi pulp with *L. fermentum* could influenced the probiotic effects of polysaccharides for the growth of *Bifidobacterium*.

**FIGURE 4:**
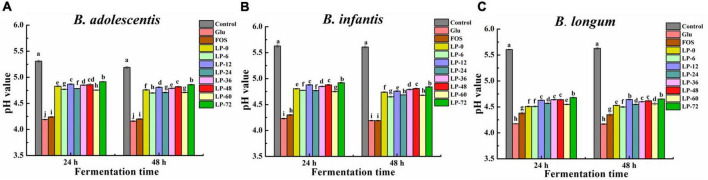
The pH of probiotic strains *Bifidobacterium adolescentis* **(A)**, *Bifidobacterium infanitis* **(B)**, and *Bifidobacterium longum* **(C)** cultivated on polysaccharides derived from litchi pulp fermented with *Lactobacillus fermentum* for different times. Mean ± SD (*n* = 3). Different letters indicate significant (*p* < 0.05) differences at the same incubation time.

The acidifying activities of probiotics, which were stimulated by LPs, FOS and Glu, were further investigated (Figures 4A–C). The pH of evaluated strains cultures decreased in the order of blank > LPs > Glu > FOS at 24 and 48 h. Among the experimental conditions for all three *Bifidobacteria*, the pH of LP-6 cultures was lower than in the LP-0 cultures but still higher than in the positive control FOS and Glu cultures. These results contrasted with the relative ordering of probiotic abundances when stimulated with polysaccharides, consistent with the results of a previous study (23). Minimum pH values reflected the acidifying activity of bacterial strains, in addition to the use of carbohydrates by the specified strain (37). Fermentation was not always promoted probiotic bacteria growth using litchi pulp polysaccharides compared with unfermented polysaccharides, contrasting with previous results for fermented longan pulp polysaccharides (19). Overall, these results indicated that a proper fermentation time by lactic acid bacteria for litchi pulp might facilitate the probiotic properties of its polysaccharides.

### Structure-function relationship

Clarifying the relationships between the structures and probiotic properties of polysaccharides was important for understanding their mechanisms of action. Previous studies have indicated that the sugar contents of polysaccharides can affect their probiotic activity (36). In this study, obvious correlations were not apparent between the contents of total sugars and uronic acids in LPs with their probiotic effects. Similar results were also observed for polysaccharides from bamboo shoot residues that were prepared with four different drying methods (38). In addition, molecular weights also influenced the probiotic effects of carbohydrates. For example, some polysaccharides with lower molecular weights are potential probiotic influencing factors (39, 40). Different ratios of monosaccharides in various fractions of rapeseed polysaccharides have been shown to contribute to their differing probiotic effects (41). Further, polysaccharides that primarily comprise glucose, xylose, and galactose exert better growth-stimulating effects (42). In the present study, LP-6 exhibited the greatest stimulation of *Bifidobacteria* growth. This could be attributed to the LP-6 sample containing the lowest average molecular weight among all LPs. Furthermore, LP-6 primarily comprised glucose, galactose, and xylose, which were the most abundant components of all LPs. In contrast, LP-48 and LP-72 cultures exhibited lower bacterial growth associated with the lower proportions of the above three components and higher Mw values. These molecular structural features interactively influenced the probiotic activities of LPs. Previous studies have observed that fermentation can be an effective means to enhance probiotic activity (19, 43). This study further observed that an appropriate fermentation time was critical for increasing probiotic activities.

## Conclusion

In this study, the impact of lactic acid bacteria fermentation on the physicochemical properties and probiotic activities of LPs was evaluated. The extraction yields, total sugar contents, uronic acid contents, molecular weights, and monosaccharide compositions of LPs varied as fermentation proceeded. Moreover, LP-6 exhibited the best probiotic activity by promoting the proliferation of *Bifidobacterium* strains. These results provide an experimental basis for the development of novel litchi polysaccharides and suggest that *Lactobacillus fermentum* fermentation can be used to generate prospective probiotic activity polysaccharides.

## Data availability statement

The original contributions presented in this study are included in the article/supplementary material, further inquiries can be directed to the corresponding authors.

## Author contributions

CH: experimental design, methodology, data curation, and writing-original draft. RZ: supervision and conceptualization. XJ, LD, QM, DZ, and ZS: supervision. MZ: conceptualization, supervision, writing-review and editing, and funding acquisition. FH: conceptualization, supervision, experimental design, writing-review and editing, and project administration. All authors listed have made a substantial, direct, and intellectual contribution to the work, and approved it for publication.

## References

[1] Zhao L Wang K Wang K Zhu J Hu Z. Nutrient components, health benefits, and safety of litchi (*Litchi Chinensis Sonn.*): a review. *Compr Rev Food Sci Food Saf.* (2020) 19:2139–63. 10.1111/1541-4337.12590 33337091

[2] Zhang Z Fan S Huang D Xiong T Nie S Xie M. Polysaccharides from Fermented *Asparagus Officinalis* with *Lactobacillus Plantarum* NCU116 alleviated liver injury via modulation of glutathione homeostasis, bile acid metabolism, and scfa production. *Food Funct.* (2020) 11:7681–95. 10.1039/d0fo01435d 32901642

[3] Wen JJ Li MZ Gao H Hu JL Nie QX Chen HH Polysaccharides from fermented *Momordica Charantia* L. With *Lactobacillus Plantarum* NCU116 ameliorate metabolic disorders and gut microbiota change in obese rats. *Food Funct.* (2021) 12:2617–30. 10.1039/d0fo02600j 33634806

[4] Wen J Ma L Xu Y Wu J Yu Y Peng J Effects of probiotic litchi juice on immunomodulatory function and gut microbiota in mice. *Food Res Int.* (2020) 137:109433. 10.1016/j.foodres.2020.109433 33233115

[5] Liu X Liu Y Chen M. Study on amino acid metabolism characteristics of litchi juice-soybean protein fermented by eight lactic acid bacteria. *Sci Technol Food Industry.* (2020) 41:106–13.

[6] Xin L Xue-Fang Z Yun L Zheng C Jie-Ping W Bo L. Dynamic of fatty acid group during fermentation of *Lactobacillus Rhamnosus* in the medium of lychee juice and soybean protein. *J Food Saf Q.* (2020) 11:3130–40.

[7] Dongwei W Yao W Haibo L Kai W Lei Z Zhuoyan H. Enhanced production of gamma-aminobutyric acid in litchi juice fermented by *Lactobacillus Plantarum* Hu-C2w. *Food Biosci.* (2021) 42:101155. 10.1016/j.fbio.2021.101155

[8] Jing Y Huang L Lv W Tong H Song L Hu X Structural characterization of a novel polysaccharide from pulp tissues of litchi chinensis and its immunomodulatory activity. *J Agric Food Chem.* (2014) 62:902–11. 10.1021/jf404752c 24320227

[9] Gao W Lin P Zeng X-A Brennan MA. Preparation, characterisation and antioxidant activities of litchi (*Litchi Chinensis Sonn.*) polysaccharides extracted by ultra-high pressure. *Int J Food Sci Technol.* (2017) 52:1739–50. 10.1111/ijfs.13447

[10] Hu XQ Huang YY Dong QF Song LY Yuan F Yu RM. Structure characterization and antioxidant activity of a novel polysaccharide isolated from pulp tissues of *Litchi Chinensis*. *J Agric Food Chem.* (2011) 59:11548–52. 10.1021/jf203179y 21973186

[11] Huang F Zhang R Dong L Guo J Deng Y Yi Y Antioxidant and antiproliferative activities of polysaccharide fractions from litchi pulp. *Food Funct.* (2015) 6:2598–606. 10.1039/c5fo00249d 26119713

[12] Huang F Liu Y Zhang R Bai Y Dong L Liu L Structural characterization and in vitro gastrointestinal digestion and fermentation of litchi polysaccharide. *Int J Biol Macromol.* (2019) 140:965–72. 10.1016/j.ijbiomac.2019.08.170 31442503

[13] Zhu W Zhou S Liu J McLean RJC Chu W. Prebiotic, immuno-stimulating and gut microbiota-modulating effects of *Lycium Barbarum* Polysaccharide. *Biomed Pharmacother.* (2020) 121:109591. 10.1016/j.biopha.2019.109591 31733576

[14] Nie Q Hu J Gao H Fan L Chen H Nie S. Polysaccharide from *Plantago Asiatica* L. Attenuates hyperglycemia, hyperlipidemia and affects colon microbiota in type 2 diabetic rats. *Food Hydrocolloids.* (2019) 86:34–42. 10.1016/j.foodhyd.2017.12.026

[15] Akbari-Alavijeh S Soleimanian-Zad S Sheikh-Zeinoddin M Hashmi S. Pistachio hull water-soluble polysaccharides as a novel prebiotic agent. *Int J Biol Macromol.* (2018) 107(Pt A):808–16. 10.1016/j.ijbiomac.2017.09.049 28928068

[16] Ouyang J Wang F Li W Li Q Su X. Structure characterization of polysaccharide from Chinese Yam (*Dioscorea Opposite Thunb.*) and its growth-promoting effects on *Streptococcus Thermophilus*. *Foods.* (2021) 10:2698. 10.3390/foods10112698 34828979 PMC8624800

[17] Huang F Hong R Zhang R Yi Y Dong L Liu L Physicochemical and biological properties of longan pulp polysaccharides modified by *Lactobacillus Fermentum* fermentation. *Int J Biol Macromol.* (2019) 125:232–7. 10.1016/j.ijbiomac.2018.12.061 30528994

[18] Azmi AF Mustafa S Hashim DM Manap YA. Prebiotic activity of polysaccharides extracted from *Gigantochloa Levis* (Buluh Beting) shoots. *Molecules.* (2012) 17:1635–51. 10.3390/molecules17021635 22314383 PMC6268289

[19] Huang F Hong R Zhang R Dong L Bai Y Liu L Dynamic variation in biochemical properties and prebiotic activities of polysaccharides from longan pulp during fermentation process. *Int J Biol Macromol.* (2019) 132:915–21. 10.1016/j.ijbiomac.2019.04.032 30959133

[20] Sevag MG Lackman DB Smolens J. Isolation of components of streptococcal nucleoproteins in serologically active form. *J Biol Chem.* (1938) 124:425–36.

[21] DuBois M Gilles KA Hamilton JK Rebers PA Smith F. Colorimetric method for determination of sugars and related substances. *Anal Chem.* (2002) 28:350–6. 10.1021/ac60111a017

[22] Chen X Zhang R Li Y Li X You L Kulikouskaya V Degradation of polysaccharides from sargassum fusiforme using Uv/H2o2 and its effects on structural characteristics. *Carbohydr Polym.* (2020) 230:115647. 10.1016/j.carbpol.2019.115647 31887897

[23] Zhang J Chen H Luo L Zhou Z Wang Y Gao T Structures of fructan and galactan from *Polygonatum Cyrtonema* and their utilization by probiotic bacteria. *Carbohydr Polym.* (2021) 267:118219. 10.1016/j.carbpol.2021.118219 34119173

[24] Chen Q Wang R Wang Y An X Liu N Song M Characterization and antioxidant activity of wheat bran polysaccharides modified by *Saccharomyces cerevisiae* and *Bacillus subtilis* fermentation. *J Cereal Sci.* (2021) 97:103157. 10.1016/j.jcs.2020.103157

[25] Wang T Ye Z Liu S Yang Y Dong J Wang K Effects of crude *Sphallerocarpus Gracilis* polysaccharides as potential prebiotics on acidifying activity and growth of probiotics in fermented milk. *LWT.* (2021) 149:111882. 10.1016/j.lwt.2021.111882

[26] Liu Q Cao X Zhuang X Han W Guo W Xiong J Rice bran polysaccharides and oligosaccharides modified by *Grifola Frondosa* fermentation: antioxidant activities and effects on the production of No. *Food Chem.* (2017) 223:49–53. 10.1016/j.foodchem.2016.12.018 28069122

[27] Jiang Y Du J Zhang L Li W. Properties of pectin extracted from fermented and steeped hawthorn wine pomace: a comparison. *Carbohydr Polym.* (2018) 197:174–82. 10.1016/j.carbpol.2018.06.001 30007603

[28] Tang N Wang X Yang R Liu Z Liu Y Tian J Extraction, isolation, structural characterization and prebiotic activity of cell wall polysaccharide from *Kluyveromyces Marxianus*. *Carbohydrate Polymers.* (2022) 289:119457. 10.1016/j.carbpol.2022.119457 35483859

[29] Yu C Ahmadi S Shen S Wu D Xiao H Ding T Structure and fermentation characteristics of five polysaccharides sequentially extracted from sugar beet pulp by different methods. *Food Hydrocolloids.* (2022) 126:107462. 10.1016/j.foodhyd.2021.107462

[30] Song C Huang F Liu L Zhou Q Zhang D Fang Q Characterization and prebiotic properties of pectin polysaccharide from *Clausena Lansium* (Lour.) skeels fruit. *Int J Biol Macromol.* (2022) 194:412–21. 10.1016/j.ijbiomac.2021.11.083 34813784

[31] Tan S Luo Z Cheng J. Effect of H2 O2-Vc degradation system on the structure and activity of polysaccharides from *Nymphaea Hybrid*. *Food Sci.* (2021) 42:48–53. 10.7506/spkx1002-6630-20201202-036

[32] Zhang W Xiang Q Zhao J Mao G Feng W Chen Y Purification, structural elucidation and physicochemical properties of a polysaccharide from *Abelmoschus Esculentus* L (Okra) flowers. *Int J Biol Macromol.* (2020) 155:740–50. 10.1016/j.ijbiomac.2020.03.235 32240742

[33] Chen J Zhang X Huo D Cao C Li Y Liang Y Preliminary characterization, antioxidant and alpha-glucosidase inhibitory activities of polysaccharides from *Mallotus Furetianus*. *Carbohydr Polym.* (2019) 215:307–15. 10.1016/j.carbpol.2019.03.099 30981359

[34] Bai Y Huang F Zhang R Dong L Jia X Liu L Longan pulp polysaccharides relieve intestinal injury in vivo and in vitro by promoting tight junction expression. *Carbohydr Polym.* (2020) 229:115475. 10.1016/j.carbpol.2019.115475 31826430

[35] Wan YJ Hong T Shi HF Yin JY Koev T Nie SP Probiotic fermentation modifies the structures of pectic polysaccharides from carrot pulp. *Carbohydr Polym.* (2021) 251:117116. 10.1016/j.carbpol.2020.117116 33142651

[36] Xing L Miao Y Li N Jiang L Chen JY. Molecular structure features and lactic acid fermentation behaviors of water- and alkali-soluble polysaccharides from *Dendrobium Officinale*. *J Food Sci Technol.* (2021) 58:532–40. 10.1007/s13197-020-04564-6 33568846 PMC7847907

[37] Gullon P Gonzalez-Munoz MJ Parajo JC. Manufacture and prebiotic potential of oligosaccharides derived from industrial solid wastes. *Bioresour Technol.* (2011) 102:6112–9. 10.1016/j.biortech.2011.02.059 21392971

[38] Chen GJ Hong QY Ji N Wu WN Ma LZ. Influences of different drying methods on the structural characteristics and prebiotic activity of polysaccharides from bamboo shoot (*Chimonobambusa Quadrangularis*) residues. *Int J Biol Macromol.* (2020) 155:674–84. 10.1016/j.ijbiomac.2020.03.223 32234437

[39] Zhao Y Bi J Yi J Wu X Ma Y Li R. Pectin and homogalacturonan with small molecular mass modulate microbial community and generate high scfas via in vitro gut fermentation. *Carbohydr Polym.* (2021) 269:118326. 10.1016/j.carbpol.2021.118326 34294338

[40] Feng R Kou J Chen S Wang N Wang W Wang L Preparation optimization, characterization, and antioxidant and prebiotic activities of carboxymethylated polysaccharides from jujube. *J Food Q.* (2021) 2021:1–15. 10.1155/2021/3268149

[41] Wang X Huang M Yang F Sun H Zhou X Guo Y Rapeseed polysaccharides as prebiotics on growth and acidifying activity of probiotics in vitro. *Carbohydr Polym.* (2015) 125:232–40. 10.1016/j.carbpol.2015.02.040 25857979

[42] Macfarlane GT Steed H Macfarlane S. Bacterial metabolism and health-related effects of galacto-oligosaccharides and other prebiotics. *J Appl Microbiol.* (2008) 104:305–44. 10.1111/j.1365-2672.2007.03520.x 18215222

[43] Lin S Wen L Yang B Jiang G Shi J Chen F Improved growth of *Lactobacillus Bulgaricus* and *Streptococcus Thermophilus* as well as increased antioxidant activity by biotransforming litchi pericarp polysaccharide with aspergillus awamori. *Biomed Res Int.* (2013) 2013:413793. 10.1155/2013/413793 23484117 PMC3581125

